# Proteomics-Based RT-qPCR and Functional Analysis of 18 Genes in Metronidazole Resistance of *Bacteroides fragilis*

**DOI:** 10.3390/antibiotics13030207

**Published:** 2024-02-22

**Authors:** Bakhtiyar Mahmood, Ana Paunkov, Malgorzata Kupc, Katalin Burián, Elisabeth Nagy, David Leitsch, József Sóki

**Affiliations:** 1 Institute of Medical Microbiology, Albert Szent-Györgyi Medical School, University of Szeged, 6725 Szeged, Hungary; mahmood.bakhtiyar@med.u-szeged.hu (B.M.); burian.katalin@med.u-szeged.hu (K.B.); nagy.erzsebet@med.u-szeged.hu (E.N.); 2 Department of Biology, University of Garmian, Kalar 2562, Kurdistan Region, Iraq; 3 Institute of Specific Prophylaxis and Tropical Medicine, Medical University of Vienna, 1090 Vienna, Austria; anna.paunkov@meduniwien.ac.at (A.P.); malgorzata.kupc@meduniwien.ac.at (M.K.); david.leitsch@meduniwien.ac.at (D.L.)

**Keywords:** *Bacteroides*, metronidazole, *nim*, resistance mechanism, RT-qPCR

## Abstract

Previously, we reported that metronidazole MICs are not dependent on the expression levels of *nim* genes in *B. fragilis* strains and we compared the proteomes of metronidazole-resistant laboratory *B. fragilis* strains to those of their susceptible parent strains. Here, we used RT-qPCR to correlate the expression levels of 18 candidate genes in a panel of selected, clinical *nim* gene-positive and -negative *B. fragilis* strains to their metronidazole MICs. Metronidazole MICs were correlated with the expression of certain tested genes. Specifically, lactate dehydrogenase expression correlated positively, whereas cytochrome fumarate reductase/succinate dehydrogenase, malate dehydrogenase, phosphoglycerate kinase redox and *gat* (GCN5-like acetyltransferase), and *relA* (stringent response) regulatory gene expressions correlated negatively with metronidazole MICs. This result provides evidence for the involvement of carbohydrate catabolic enzymes in metronidazole resistance in *B. fragilis*. This result was supported by direct substrate utilization tests. However, the exact roles of these genes/proteins should be determined in deletion–complementation tests. Moreover, the exact redox cofactor(s) participating in metronidazole activation need to be identified.

## 1. Introduction

Species within the former *Bacteroides fragilis* group (BFG) (which are now classified within the *Bacteroides*, *Parabacteroides*, and *Phocaeicola* genera) are the most frequently isolated opportunistic anaerobic pathogens, which are also important members of the mammalian intestinal microbiota. Of these, *B. fragilis* is the most pathogenic and accounts for 50% of clinical isolates; however, it is only a minor population among intestinal strains [1]. They are highly resistant to most antimicrobial agents through the use of several antibiotic resistance mechanisms. Metronidazole is a prominent choice to treat infections of *B. fragilis* because metronidazole is an anti-anaerobic drug that usually elicits low levels of resistance among obligately anaerobic pathogens [2]. However, metronidazole resistance levels among *Bacteroides* have increased somewhat and increased greatly in developed and developing countries, respectively [3,4]. The best known and most investigated metronidazole resistance mechanism of BFG strains is mediated by the *nim* genes, a few of which were originally discovered in the 1980s and 1990s [5]. There are now 12 known homologs of *nim* (i.e., *nimA-L*) that share 50–80% amino acid homologies, and they are all proposed to act as nitro-reductases [6,7]. They have been localized to both the plasmid and chromosome, and they all require an upstream copy of a *Bacteroides*-specific insertion sequence (IS) element with promoter sequences to function in metronidazole resistance [5]. Besides *nim* genes, the other proposed metronidazole resistance mechanisms among BFG strains include increased lactate dehydrogenase, decreased pyruvate-ferredoxin oxidoreductase (PFOR), efflux, reduced iron uptake, and increased DNA repair [5]. However, these latter diverse mechanisms do not operate in all *nim*-negative-resistant strains and are sometimes found only in laboratory strains after the induction of metronidazole resistance. The *nim*-mediated mechanism, which is most prevalent among *Bacteroides*, still has some open questions. However, it is known that the metronidazole MICs of *nim*-positive strains are sometimes low and unstable, and the expression level of the *nim* genes correlates poorly with metronidazole MICs, which tend to be flexible. To explain this low correlation, the action of auxiliary factors has been proposed [8].

*B. fragilis* strains can be classified into two divergent divisions based on genetic differences (e.g., differences in alleles or genetic elements, most importantly the carbapenem resistance gene, *cfiA*) that can be mapped to specific loci in the genome of this important species [9], and, by our observations, it is also related to the gene expressions of the *B. fragilis* strains.

The aim of this study was to search for factors, in addition to *nim* genes, that affect the metronidazole resistance of *B. fragilis*. We have previously revealed that hemin- and iron-uptake mechanisms are involved in metronidazole resistance, and *nim*-negative and *nim*-positive *B. fragilis* strains behave differently in these regards [10]. Previously, we analyzed the proteomics of *nimA*-positive and -negative *B. fragilis* laboratory strains [11]. In addition, here, we analyzed the expression of 18 genes previously identified as resistance candidates in a proteomic study in a collection of *nim*-positive and -negative clinical *B. fragilis* strains, and we correlated the expression levels of these genes to measurements of metronidazole MICs to identify possible auxiliary factor(s) involved in metronidazole resistance of *B. fragilis*. Finally, we studied the effects of C_4_-dicarboxylic acid supplementation on metronidazole MICs to better understand the hemin dependence of metronidazole resistance.

## 2. Results and Discussion

### 2.1. Connection between the Metronidazole MICs and nim Gene Expression

Table 1 shows the results of the metronidazole MICs and the expression levels of the *nim* genes of the eight *nim*-positive strains. Similarly to the results of our previous studies [8,11], metronidazole MICs and the expression levels of the *nim* genes were independent of each other (r = 0.185, *p* = 0.619, r^2^ = 0.0342).

However, *nim* genes are known resistance factors because they transfer the resistance phenotype in conjugation experiments [19,20,21], and they are associated with metronidazole resistance in field studies [4,22]. Therefore, there is a need to account for this lack of correlation even with the same *nim* gene and IS element pairs. Previously, we proposed the existence of rate-limiting factors that influence the metronidazole resistance of *B. fragilis* strains [8].

### 2.2. Examination of the Roles of 18 Genes in Metronidazole Resistance

To investigate this possibility, we measured the expression levels of 18 genes selected according to the results of previous research or from our recent investigations [11] using RT-qPCR to study 15 *B. fragilis* strains. The cross-correlations between gene expressions and the correlation between gene expression and metronidazole MICs for all 15 *B. fragilis* strains are shown in Table 2. The cross-correlations between certain genes were very strong (r > 0.7, *p* < 0.01), indicating their common regulation, although not all genes (except for *frdA* and *frdC*, whose expressions correlated well—r = 0.593, *p* = 0.0192, Table 2) are located on the same operon [23]. Moreover, we detected highly significant correlations between the expression of some genes and metronidazole MICs. In particular, lactate dehydrogenase (*ldh*) expression correlated positively, whereas cytochrome b fumarate reductase/succinate dehydrogenase (*frdC*), malate dehydrogenase (*mdh*), phosphoglycerate kinase (*pgk*) catabolic and *gat* (GCN5-related acetyltransferase toxin), and *relA* (stringent response regulator) regulatory gene expressions correlated negatively with metronidazole MICs. Within the *nim*-positive and *nim*-negative strains, we detected cross-correlations between gene expressions; however, we found no significant association between metronidazole MICs and gene expressions, except for *mdh* and *gat*, which tended to correlate with metronidazole MICs in the *nim*-positive and *nim*-negative groups (Tables S2 and S3), respectively. In addition, the gene cross-correlations of the full set did not overlap with those in the *nim*-positive and *nim*-negative groups of strains (*cf.* Table S2 and Table 3). The lack of statistical confirmation may be due to the low number of strains in each group (eight *nim*-positive and seven *nim*-negative strains).

However, one-way variance analysis (Table 3) demonstrated that *frdC*, *gat*, *mdh*, *nanH* (sialidase), *pgk*, and *relA* gene expression depended on the presence of the *nim* gene; however, the *cfiA* gene status did not affect the expression of the studied genes (Table 3). The genes listed above differ from the list in Table 3 because the list above includes and excludes *ldh* and *nanH*, respectively. We are currently unable to explain this finding, although the inclusion of *nanH* indicates a link between metronidazole resistance/*nim* positivity and virulence.

Although we found no significant association of the examined genes among the *nim*-negative and *nim*-positive strains separately, the combined data signalized some good associations in the case of the whole strain set (see above). Therefore, we conclude that no particular enzyme is exclusively correlated with metronidazole resistance in both the *nim*-negative and -positive strains. However, some of these genes have previously been found to cause metronidazole resistance, e.g., *feoAB* (described in [24]), *acr5* (*bmeB*, described in [25]) and *por* (described in [26]). This may be applied to genes not examined here (*recA*, *sod* and *rhaA*), as their role has been demonstrated in metronidazole resistance earlier [27,28,29]. So, on the population level, these genes do not exert a general role; they are important only in individual cases/strains. However, it should be noted that in both *nim*-positive and *nim*-negative strains, the possible exceptions of *mdh* and *gat* could be significant, respectively (mentioned above).

In addition, the roles of enzymes involved in the central metabolism in *B. fragilis* should also be considered. The central metabolism varies greatly among bacteria [30], e.g., the central metabolism of *Bacteroides* differs greatly from that of γ-Proteobacteria, and the latter comprises glycolysis and parts of the tricarboxylic cycle (TCA). However, instead of a complete TCA cycle, *Bacteroides* have a reductive or reverse TCA (rTCA) branch that is heme dependent, as well as a branch that is heme independent (Figure S1) [31]. Previously, we found that hemin depletion causes metronidazole susceptibility in both *nim*-negative and *nim*-positive strains of *B. fragilis* [10]. Thus, heme may be a rate-limiting factor in the metronidazole resistance of *B. fragilis*, as proposed above. Our results show that the expression of genes from the glycolytic and rTCA pathways (*pgk*, *frdC*, and *mdh*) correlate negatively with the metronidazole MICs, whereas that of *ldh* correlates positively. These latter changes can decrease the cellular concentrations of reducing cofactor, which diminishes metronidazole activation, thus inducing resistance.

The Nim enzymes are nitro-reductases that can transfer either six [6] or two electrons to the nitro group of metronidazole, yielding either an amino or a nitroso imidazole, respectively [20]. Recently, in vivo and in vitro experiments demonstrated that a *nim* group enzyme encoded by *Clostridioides difficile* strains is a nitro-reductase [7]. In this latter study, it was also confirmed that metronidazole resistance in *C. difficile* is dependent on hemin [32] through experiments involving the direct addition of metronidazole to assay its modification by recombinant NimB and by transcriptomic analysis. Moreover, genetic (transposon mutagenesis) and biochemical (aromatic nitro-reduction to amine) tests have proven that the *nimB* gene of some *C. difficile* strains is responsible for their metronidazole resistance. However, in the in vitro experiments, the metronidazole concentration used, 5 mM, was much higher than that to which the bacteria are usually exposed (the 4 μg/mL breakpoint concentration corresponds to 23.4 μM—a ca. 160-fold difference). It is possible that the hemin dependence of metronidazole resistance is due to the hemin dependence of the NimB protein; however, this does not explain the hemin dependence of *nim*-negative strains.

### 2.3. Examination of Addition of C_4_-dicarboxylic Acids on Metronidazole Resistance

We were also interested in how the addition of intermediates of the rTCA pathway affects metronidazole MICs. We expected that higher oxaloacetate or fumarate concentrations would decrease the redox intermediate concentration (e.g., NADH), thus decreasing metronidazole activation and MICs. In these experiments, we used modified M9 minimal medium supplemented with Tryptone, hemin, vitamin K1, and glucose or C_4_-dicarboxylic acid. The results are shown in Table S4, with some representative plates shown in Figure S2. Out of six *nim*-negative or *nim*-positive strains, four showed no significant difference in metronidazole MICs compared to those obtained on supplemented Columbia agars. However, the MICs of one *nim*-positive and one *nim*-negative strains increased in response to glucose, malate, and succinate addition, whereas no changes were observed in response to oxaloacetate or fumarate addition. This latter finding supports our assumption that is noted above (e.g., that the metronidazole resistance is highly dependent on reducing cofactor(s)). Moreover, our findings are consistent with the previous observation on the flexibility of metronidazole MICs and the idea of a rate-limiting step(s) involved in *nim* action in metronidazole resistance. We propose the following mechanism: the addition of malate and succinate forces the cells to reduce the levels of these compounds at the expense of the pool of reducing cofactors, thus leading to decreased metronidazole activation. We also argue that the C_4_-dicarboxylic acid uptake rates probably do not affect these processes because one transport protein, the anaerobic C_4_-dicarboxylic carrier protein, is responsible for their uptake with similar efficiencies in *Escherichia coli* [33]. The ortholog of this carrier protein is present in the genomes of *B. fragilis* strains (our unpublished analysis). The observed increase in *ldh* gene expression is consistent with previous findings, showing the importance of reducing cofactors in metronidazole resistance in anaerobic bacteria [26]. This means that the pyruvate level is the main mediator in this latter process. However, we did not observe a differential expression of *por* during our experiment. It is possible that withdrawing hydrogen/reducing cofactors from metronidazole activation may be involved in this process. The involvement of *frdC* (a cytochrome b enzyme) in metronidazole resistance is noteworthy because it can explain, at least partly, the heme dependence of the metronidazole resistance of *B. fragilis.* Additionally, the negative correlation of the regulatory genes (*relA* and *gat*) suggests that a high metabolic state is required for metronidazole to act on cells because these genes have a role in decreasing cellular metabolism.

This study is the first to examine the role of multiple proteins/genes on metronidazole resistance in clinical *B. fragilis* strains. Earlier modeling studies have focused only on laboratory strains of *B. fragilis*. For example, based on the roles of a limited set of proteins analyzed by two-dimensional protein electrophoresis and northern blotting, Diniz et al. proposed that *ldh* and *por* participate in metronidazole activation at certain low levels [26,34]. However, their model was not confirmed by Paunkov et al. [35]. Also, de Freitas et al. analyzed the transcriptome-wide effect of metronidazole on a large number of proteins, and they confirmed that, along with some other proteins, the concentration of activating ferredoxin is important in alleviating metronidazole stress [36]. Based on the results of proteomic studies, Paunkov et al. developed models of how *nim* and other proteins act in *nim*-dependent and -independent metronidazole-resistant *B. fragilis* strains [11].

### 2.4. Proposal for the Interactions of Redox and Other Proteins in Metronidazole Resistance

**Figure 1:**
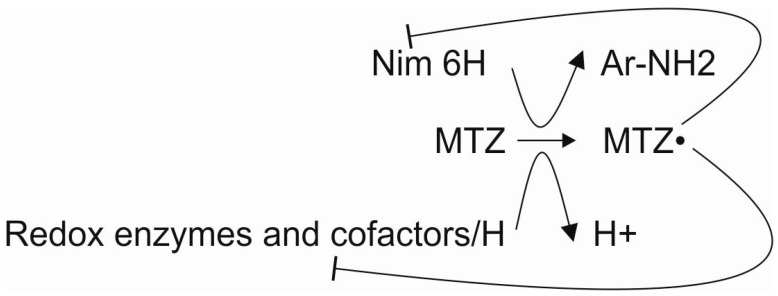
Interactions of the participants in metronidazole resistance. Lines with the ͱ symbol mean inhibition.

Here, we propose that a limited number of genes/proteins are correlated with metronidazole resistance in *B. fragilis* at the population level. In this study, we highlight the importance of reducing cofactors that are needed for both metronidazole activation and inactivation. The activated metronidazole radical acts by reducing *nim* and redox cofactor proteins and thiol compounds of the proteome [37]. Thus, the metronidazole resistance mechanism of *B. fragilis* is complex and nonlinear. This complexity can explain why metronidazole MICs and *nim* gene expression do not always correlate, especially long after the isolation of strains from clinical specimens. Thus, the process of developing resistance to metronidazole is also complex (Figure 1).

Earlier work suggested that ferredoxin is responsible for reducing metronidazole [26]; however, we were unable to find a role for PFOR (negative association), as we observed increased PFOR and PFOR activities in laboratory metronidazole-resistant *B. fragilis* strains [35]. Thus, more work is needed to determine significant associations between gene expression and metronidazole MICs in “field” strains *nim*-negative and -positive *B. fragilis*. In particular, more strains need to be analyzed to prove the roles of those genes. In addition, the roles of the genes that had positive or negative correlations with metronidazole resistance should be confirmed by deletion–complementation analysis. In particular, *frdC* is a good candidate for these experiments because it also contains heme. Identifying with more certainty which redox cofactor activates metronidazole also remains a future task. The starting point for this study was a proteomic analysis of metronidazole resistant laboratory strains [11], but only some of them proved to be effective in metronidazole resistance on a population level; therefore, we believe that the genes that had a role in our study (*ldh*, *frdC, mdh*, *pgk, gat* and *relA*) are significant/valid contributors in this important kind of antibiotic resistance mechanism.

## 3. Materials and Methods

### 3.1. Bacterial Strains and Cultivation

Sixteen *B. fragilis* test strains (Table 1 and Table S1) with known genetic backgrounds were stored in 20% glycerol stocks at −80 °C and cultivated on supplemented Columbia blood agar medium (SCA, Columbia base, supplemented with 2.5% defibrinated sheep blood, 0.5% laked sheep blood, 0.3 mg/mL L-cysteine, 1 µg/mL vitamin K_1_) or in supplemented brain–heart infusion broth (BHIS, brain–heart infusion broth supplemented with 2.5% yeast extract, 10 µg/mL hemin and 1 µg/mL vitamin K_1_) under anaerobic conditions (85% N_2_, 10% H_2_, 5% CO_2_, Concept 400 anaerobic cabinet (Ruskinn, Bridgend, UK)) at 37 °C. The strains include both *nim*-positive and -negative *B. fragilis* strains whose *cfiA* gene statuses are known (Table 1). Parallelly with metronidazole MIC determinations, we used the same SCA plates for inoculations of 5 mL of BHIS for RNA isolation and cell suspensions to determine MICs. To test the effect of C_4_-dicarboxylic acids on metronidazole resistance, we used a semi-defined M9-based agar medium (48 mM Na_2_HPO_4_, 22 mM KH_2_PO_4_, 8.5 mM NaCl, 1.6 mM NH_4_Cl, 2 mM MgSO_4_, 0.1 mM CaCl_2_, 1% casein peptone Type I (Neogene), 0.625% yeast extract, 10 mM glucose or 15 mM C_4_-dicarboxylic acid (oxaloacetate/D(-) malate/fumarate/succinate), 10 µg/mL hemin, 1 µg/mL vitamin K_1_) to perform MIC measurements.

### 3.2. Metronidazole MIC Measurements

Metronidazole MICs were measured using a gradient method (Etest, bioMérieux, Hungary Ltd., Budapest, Hungary). First, McFarland density suspensions were made in a phosphate-buffered saline solution (137 mM NaCl, 2.7 mM KCl, 100 mM Na_2_HPO_4_ and 1.8 mM KH_2_PO_4_, pH 7.4), with which we inoculated the surface of SCA plates by cotton swabs, and applied the Etest strips, and after anaerobic cultivation at 37 °C for 48 h, we read the plates.

### 3.3. RT-qPCR

We extracted total RNA from 5 mL BHIS cultures for RT-qPCR experiments using the HighPure RNA Isolation Kit (Roche). The quantity and quality of RNA were assessed using the Qubit 4 fluorometer and the Qubit RNA BR and RNA Integrity kits (Thermo Fisher Scientific, Waltham, MA, USA). Of 32 candidate genes identified in previous proteomic studies [11], we chose 18 and designed primer pairs using the Primer3 Plus software (www.primer3plus.com). During primer design, we took into account the possibility that the *cfiA*-positive and -negative strains may differ in their respective sequences. Therefore, the consensus nucleotide sequences of the selected genes were obtained from the complete genomic sequences of the *cfiA*-negative and -positive strains *B. fragilis* 638R (GenBank Acc. No. NC_016776) and *B. fragilis* 3130 (GenBank Acc. No. LJVI01), respectively, to design primer pairs. We used the *gap*, *rrn*, and *rpoD* genes as endogenous controls. The nucleotide sequences of the primers used are shown in Table S1. The 10 μL RT-qPCR reactions contained 5.6 μL kit components (Verso 1-step SYBR RT-PCR mastermix, Thermo Fisher Scientific), 0.2 μL each primer (35 μM), 3 μL H_2_O, and 1 μL total RNA. The reactions were incubated in an RT-PCR instrument (QuantStudio 3, Thermo Fisher Scientific) in 100 μL 96-well plates using the following conditions: 35 cycles consisting of 55 °C 20 min, 95 °C 15 min; 95 °C 15 s, 55 °C 30 s, 72 °C 30 s. The melting curves were recorded using 3 technical replicates. We detected the expression of the *nim* genes in 8 *nim*-positive *B. fragilis* strains by amplifying *nim* PCR products using the same conditions as those described above, except the 35 PCR cycles consisted of two steps (55 °C 20 min, 95 °C 15 min; 95 °C 15 s, 60 °C 1 min; melting curve) because three *nim* gene types were included.

### 3.4. Data Analysis

We used the amplification threshold values (C_T_) from RT-qPCR experiments to calculate the ratios of gene expression by the ΔΔC_T_ method. The calculations were performed by the Relative Quantitation App on the Thermo Fisher Scientific webpage (www.thermofisher.com). One-way variance (ANOVA), Spearman’s rank, and cross-correlation values were calculated using SigmaPlot 12 software (Sigmaplot, Erkrath, Germany).

## 4. Conclusions

In this study, we assessed the connection between metronidazole MICs and the expression of 18 genes in a wide selection of *B. fragilis* clinical strains. The expression of metabolic genes *ldh*, *frdC, mdh*, and *pgk* correlated with metronidazole resistance independently of the presence of *nim* genes. This finding emphasizes that redox intermediates may be crucial in both metronidazole activation and enzymatic inactivation. However, the exact identities of the enzymes and intermediates involved in both processes need to be confirmed experimentally. Roles for some regulatory proteins (*gat*, *relA*) were also found and not all (genes)/proteins could be examined here as they were differentially expressed at the protein level. Thus, the list of examined genes should also be increased.

## Figures and Tables

**Table 1:** Strains used, their properties and RT-qPCR experiment results.

*B. fragilis*	MTZ ^a^ MIC (µg/mL)	*nim* (IS)	*nim* experession (Rq ^b^)	*cfiA*	Ref.
GBR13	>256	E (IS*Bf6*)	0.352	+	[12]
388/2	>256	E (IS*Bf6*)	1.778	+	[13]
Q5	256	E (IS*Bf6*)	1.411	+	[14]
20584	256	E (IS*Bf6*)	1	+	This study
Q6	256	E (IS*Bf6*)	0.187	-	[14]
DOR18i3	256	D (IS*1169*)	0.403	+	This study
18807i2	(0.5−) > 256 ^c^	-	n.a.	-	This study
Q11	64	E (IS*Bf6*)	0.856	+	[14]
WI1	32	-	n.a.	+	[15]
KSB-R	32	B (IS*1186*)	0.109	+	[16]
SY46	0.25	-	n.a.	-	[17]
SZ69	0.25	-	n.a.	+	[17]
638R	0.125	-	n.a.	-	[18]
SZ26	0.125	-	n.a.	+	[17]
SE61	0.064	-	n.a.	-	[17]

^a^ MTZ stands for metronidazole. ^b^ Rq is relative quantity determined by the ΔΔC_T_ method. ^c^ Heterogeneous resistance phenotype.

**Table 2:** Cross-correlation values between the examined gene expressions and the metronidazole MICs for 15 *B. fragilis* strains ^a^.

	S3	acr5	*acr15*	*crpF*	*frdC*	*feoAB*	*fldA*	*fprA*	*frdA*	*galK*	*gatMZ*	*ldh*	*mdh*	*nanH*	*porMZ*	*pgk*	*relA*	MIC ^b^
L20	0.486	−0.318	0.243	−0.346	0.725	0.0393	−0.479	0.421	0.789	0.154	0.149	−0.0964	0.621	−0.393	0.257	0.704	0.679	−0.423
	0.0639	0.24	0.374	0.199	0.00178	0.883	0.0685	0.113	2 × 10^−7^	0.575	0.584	0.724	0.0129	0.142	0.346	0.00302	0.00504	0.113
S3		0.214	0.429	−0.296	0.511	−0.175	0.025	0.275	0.443	0.579	0.31	0.0286	0.421	−0.321	0.414	0.643	0.614	−0.25
		0.433	0.107	0.275	0.0498	0.523	0.923	0.312	0.0946	0.0231	0.252	0.913	0.113	0.235	0.12	0.00934	0.0143	0.359
*acr5*			0.461	0.136	−0.343	−0.113	0.393	0.05	−0.132	0.486	−0.125	0.211	−0.111	0.25	0.443	−0.0536	−0.168	0.227
			0.0808	0.62	0.204	0.676	0.142	0.852	0.629	0.0639	0.648	0.441	0.686	0.359	0.0946	0.842	0,54	0.41
*acr15*				0.4	−0.0179	−0.0536	0.0821	0.307	0.546	0.629	−0.133	0.664	0.0107	0.343	0.639	0.125	0.104	0.132
				0.134	0.944	0.842	0.763	0.257	0.0339	0.0116	0.629	0.00654	0.964	0.204	0.00988	0.648	0.705	0.629
*crpF*					−0.3	0.211	0.0929	−0.214	0.0429	0.075	−0.262	0.646	−0.25	0.468	0.439	−0.368	−0.25	0.491
					0.269	0.441	0.734	0.433	0.873	0.783	0.339	0.00882	0.359	0.0757	0.0975	0.171	0.359	0.0597
*frdC*						0.179	−0.336	0.486	0.593	−0.193	0.528	−0.311	0.7	−0.614	0.25	0.75	0.814	−0.669
						0.514	0.214	0.0639	0.0192	0.481	0.0413	0.252	0.00326	0.0143	0.359	0.000786	2 × 10^−7^	0.00614
*feoAB*							0.259	0.45	−0.132	−0.37	−0.0403	0.247	0.182	−0.316	0.39	−0.218	0.261	−0.0118
							0.339	0.0889	0.629	0.167	0.883	0.367	0.506	0.24	0.146	0.426	0.339	0.964
*fldA*								−0.05	−0.536	0.118	−0.0685	0.468	−0.207	0.286	0.143	−0.386	−0.0429	0.274
								0.852	0.0382	0.667	0.802	0.0757	0.449	0.293	0.602	0.15	0.873	0.312
*fprA*									0.218	−0.179	0.27	−0.0536	0.575	−0.454	0.386	0.461	0.471	−0.426
									0.426	0.514	0.319	0.842	0.0241	0.0861	0.15	0.0808	0.0732	0.11
*frdA*										0.279	0.157	0.143	0.393	−0.1	0.357	0.55	0.489	−0.361
										0.306	0.566	0.602	0.142	0.714	0.185	0.0325	0.0618	0,18
*galK*											−0.475	0.482	−0.25	0.382	0.339	0.0643	−0.075	0.457
											0.0708	0.0662	0.359	0.154	0.209	0.812	0.783	0.0834
*gatMZ*												−0.5	0.596	−0.58	−0.00403	0.463	0.483	−0.683
												0.0556	0.0183	0.0231	0.985	0.0782	0.0662	0.00471
*ldh*													−0.3	0.564	0.421	−0.346	−0.0893	0.517
													0.269	0.0275	0.113	0.199	0.743	0.0463
*mdh*														−0.689	0.364	0.836	0.825	−0.528
														0.00409	0.176	2 × 10^−7^	2 × 10^−7^	0.0413
*nanH*															−0.0214	−0.521	−0.543	0.446
															0.934	0.0446	0.0353	0.0917
*porMZ*																0.254	0,45	0.0798
																0.353	0.0889	0.773
*pgk*																	0.786	−0.586
																	2 × 10^−7^	0.0211
*relA*																		−0.629
																		0.0116

^a^ Odd and even rows with correlation coefficients and significance values, respectively; the color-coding means the following: yellow—0.5 < r < 0.7, 0.05 > *p* >0.01, orange: r > 0.7, *p* < 0.01; abbreviations in Table S1. ^b^ Metronidazole MIC.

**Table 3:** Significances of the one-way variance analyses of the gene expressions and the metronidazole MICs depending on the genetic background in 15 *B. fragilis* strains.

	L20	S3	*acr5*	*acr15*	*crpF*	*frdC*	*feoAB*	*fldA*	*fprA*	*frdA*	*galK*	*gat*	*ldh*	*mdh*	*nanH*	*por*	*pgk*	*relA*	MIC
*nim*	n.s.^a^	n.s.	n.s.	n.s.	n.s.	0.006	n.s.	n.s.	n.s.	n.s.	n.s.	0.029	n.s.	0.001	0.04	n.s.	0.001	0.001	0.001
*cfiA*	n.s.	n.s.	n.s.	n.s.	n.s.	n.s.	n.s.	n.s.	n.s.	n.s.	n.s.	n.s.	n.s.	n.s.	n.s.	n.s.	n.s.	n.s.	n.s.

^a^ n.s.—non-significant.

## Data Availability

The datasets of this study are available from the corresponding author upon reasonable request.

## Supplementary Materials

The following supporting information can be downloaded at: https://www.mdpi.com/article/10.3390/antibiotics13030207/s1 Table S1. RT-qPCR target genes and primer sequences. Table S2. Cross-correlation values of gene expressions metronidazole resistance for eight *nim*-positive *B. fragilis* strains. Table S3. Cross-correlation values of gene expressions metronidazole resistance for seven *nim*-negative *B. fragilis* strains. Figure S1. The tricarboxylic acid pathways of *B. fragilis*. Figure S2. Examples of the Etest results on modified M9 medium. Table S4. Effects of C_4_ dicarboxylic acid supplementation on metronidazole MICs of selected *nim*-positive and negative *B. fragilis* strains.
