# Supplementary material for: Proteomics-Based RT-qPCR and Functional Analysis of 18 Genes in Metronidazole Resistance of Bacteroides fragilis
Source: Antibiotics (Basel). 2024 Feb 22;13(3):207. doi: 10.3390/antibiotics13030207 (PMC10967509; doi:10.3390/antibiotics13030207)
Supplement: Supplementary file 1 [file antibiotics-13-00207-s001.zip › antibiotics-2852170-supplementary.pdf]

Supplementary material

Table S1. RT-qPCR target genes and primer sequences

	Gene id. Bf 638R	MS[a] FC	Ref.	Description/Name	Cellular pathway[b]	Primer designation	sequence F(1)[d]	sequence R(2)[d]
1	BF638R_0963	8,30	[24]	FprA family A-type flavoprotein (*fprA*)	1	fprA1/2	TAACGACCGTAACANGCACC	GATCAGTGCCACCATTTCAT
2	BF638R_1663	10,55	[24]	galactokinase (*galK*)	1	galK1/2	TGGCAGANGCAAAAGCTGAT	YTCGTAAAGTTTGCTCATGC
3	BF638R_1728	-2,95	[24]	exo-alpha-sialidase (*nanH*)	2	nanH1/2	TGTGTGCTATACCGGTAGTT	GTCTGGCTCTCTTTAGCATC
4	BF638R_3176	8,40	[24]	BmeB15 (*acr15*)	2	act15-1/2	AGTATCGATCACCTTCGGAA	TACTTGCTGTCGTTCAGTTC
5	BF638R_3999	9,10	[24]	BmeB5 (*acr5*)	2	acr51/2	CCTATCGACTTGTATCCGGA	ATTCAGCGTATTCTCCAACG
6	BF638R_2281	4,00	[24]	RelA/SpoT family protein (*relA*)	5	relA_1/2	CGTGACCGTTATATTGCCAA	ACTGGCATTTCTGTTTCTTCA
7	BF638R_1701	22,80	[24]	50S ribosomal protein L20	4	L20_31/2	CGTGACCGTAGAAACAAGAA	AAGTCAGCCAAAACCTTACG
8	BF638R_4035	15,10	[24]	30S ribosomal protein S3	4	S3-1/2	GTGGCCAGGAAGTTGATAAG	ACAATCACAGCATCCAGTTC
9	BF638R_3828	-	This study	malate dehydrogenase (*mdh*)	1	mdh-1/2	GACTCGTGAAGAACTGATCG	AGTCATTGTATCCATCGGGT
10	BF638R_1473	-	[17]	lactate dehydrogenase (*ldh*)	1	ldh-1/2	TCGGACCGTATCATNGACGA	TATTCTCCAGAGTCGTTGCT
11	BF638R_3133	-	This study	phospho-glycerate kinase (*pgk*)	1	pgk-1/2	TTCGAAAACTTCACTCACGG	TACCCGGAAGAACTTTTCCT
12	BF638R_1421	-	[16]	*feoAB*	3	feoAB-1/2	GCTTCCGTAAACGNATTGTG	GTCGNCGCAGAGAGATTTCA
13	BF638R_2696	4,1	[24]	flavodoxin (*fldA*)	1	fldA-1/2	ATTTTACGGTTCCACAACGG	TCACTAACTCGTCGTTCAGT
14	BF638R_4500	-	This study	fumarate reductase (*frdA*)	1	frdA-1/2	ATCTGGGTTCCGAAGAAGAT	GCGTTCCAGATAGAAGTCAC
15	BF638R_4499	11,50	[24]	succinate dehydrogenase/fumarate reductase cytochrome b subunit (*frdC*)	1	frdC-1/2	TGGGCTAAAATGCAGTTACC	CAGAAACCATGAGTCAGGTG
16	BF638R_2263	2,40	[24]	Crp/Fnr family transcriptional regulator (*crpF*)	5	crpF-1/2	CGTGATTTTGACGGAATCG	TAATCGATCATTTCTCCCCG
17	BF638R_1544	-3,20	[24][c]	GNAT family N-acetyltransferase (*gat*)	5	gatMZ-1/2[c]	TTCCCTATCGTAGCATACCC	TCTGGTGTCCACTCTGTATC
18	BF638R_3194	43,12	This study[c]	pyruvate-flavodoxin/ferredoxin oxidoreductase (*por*)	1	porMZ-1/2[c]	ATGACATTCCGTATCCAGGT	GTTGTTTGCTCTTCACGTTG
	Endogeneous controls							
19	BF638R_0945		This study	glyceraldehyde-phosphate dehydrogenase (*gap*)	-	gap1/3BF	AGCCATTGTAGCAGCTTTTT	GAAGACGGGATGATGTTTC
20	6 16S rRNA		This study	16S rRNA (*rrn*)	-	rrnBF-1/2	TCCTGTTTGATACCCACACT	GCTCAACCGTAAAATTGCAG
21	BF638R_2767		This study	*rpoD*	-	rpoD_BF1/2	CCAATCTTCGTTTCGTCGTA	TTCATCAAACTTCTCGGCAG

[a] FC means fold change in the proteomic study. [b] The numbers designate the following pathways - 1: glycolysis, carbohydrate metabolism, redox processes, 2: cell-wall, efflux, virulence, 3: heme/Fe2+ metabolism, 4: protein synthesis, 5: nucleic acids, repair, stress, gene regulation. [c] These items were selected from the different paralog genes of these proteins in the B. fragilis genomes based on differential expressions, the pyruvate-ferredoxin oxidoreductase gene here was selected on its differential expression in RNASeq experiments (our unpublished results). [d] Forward (1) and reverse (2) orientations.

Table S2. Cross-correlation values of gene expressions metronidazole resistance for eight *nim*-positive *B. fragilis* strains[a]

	S3	*acr5*	*acr15*	*crpF*	*frdC*	*feoAB*	*fldA*	*fprA*	*frdA*	*galK*	*gatMZ*	*ldh*	*mdh*	*nanH*	*porMZ*	*pgk*	*relA*	MIC[b]
L20	0,476	0,429	0,762	-0,167	0,31	-0,333	-0,214	0,286	0,881	0,762	-0,247	0,143	0,119	0,429	0,381	0,5	0,357	-0,334
	0,207	0,26	0,0212	0,662	0,423	0,387	0,578	0,46	2E-07	0,0212	0,537	0,705	0,749	0,26	0,321	0,182	0,353	0,387
S3		0,548	0,333	-0,333	0,548	0,143	0,357	0,619	0,0952	0,738	0,577	-0,119	0,429	0,0238	0,667	0,595	0,81	-0,124
		0,139	0,387	0,387	0,139	0,705	0,353	0,086	0,794	0,0287	0,12	0,749	0,26	0,931	0,0588	0,102	0,0096	0,749
acr5			0,286	-0,0476	0,119	-0,333	-0,262	0,476	0,143	0,452	0,412	-0,429	0,857	-0,19	0,714	0,81	0,262	0,272
			0,46	0,885	0,749	0,387	0,498	0,207	0,705	0,233	0,29	0,26	0,00178	0,619	0,0374	0,0096	0,498	0,498
acr15				0,0238	0,143	-0,429	0,0238	0,5	0,81	0,5	-0,247	0,476	0,19	0,786	0,262	0,357	0,119	-0,136
				0,931	0,705	0,26	0,931	0,182	0,0096	0,182	0,537	0,207	0,619	0,0149	0,498	0,353	0,749	0,705
crpF					0	-0,619	-0,119	-0,452	0,0476	0,0952	-0,577	0,333	0,214	0,381	0,0952	0,286	0	0,568
					0,977	0,086	0,749	0,233	0,885	0,794	0,12	0,387	0,578	0,321	0,794	0,46	0,977	0,12
frdC						0,0238	-0,0476	0,429	0,262	0,476	0,0825	-0,167	-0,167	0,0476	0,167	0,452	0,405	-0,507
						0,931	0,885	0,26	0,498	0,207	0,839	0,662	0,662	0,885	0,662	0,233	0,29	0,182
feoAB							0,619	0,0714	-0,452	-0,357	0,577	0,0476	-0,357	-0,548	-0,5	-0,619	0,0476	-0,148
							0,086	0,839	0,233	0,353	0,12	0,885	0,353	0,139	0,182	0,086	0,885	0,705
fldA								0,0952	-0,333	0,0238	0,412	0,595	0,0238	0,0952	-0,119	-0,333	0,429	0,309
								0,794	0,387	0,931	0,29	0,102	0,931	0,794	0,749	0,387	0,26	0,423
fprA									0,167	0,167	0,577	-0,19	0,286	0,0952	0,286	0,357	0,0714	-0,321
									0,662	0,662	0,12	0,619	0,46	0,794	0,46	0,353	0,839	0,423
frdA										0,5	-0,577	0,333	-0,143	0,619	0,0476	0,286	0	-0,383
										0,182	0,12	0,387	0,705	0,086	0,885	0,46	0,977	0,321
galK											-0,0825	0,0714	0,333	0,381	0,738	0,738	0,833	-0,0865
											0,839	0,839	0,387	0,321	0,0287	0,0287	0,00526	0,794
gatMZ												-0,412	0,412	-0,577	0,247	0,0825	0,247	0,0856
												0,29	0,29	0,12	0,537	0,839	0,537	0,794
ldh													-0,19	0,667	-0,333	-0,333	0,0714	0,272
													0,619	0,0588	0,387	0,387	0,839	0,498
mdh														-0,0714	0,738	0,69	0,31	0,667[c]
														0,839	0,0287	0,0474	0,423	0,0588
nanH															0,143	0,143	0,119	-0,0618
															0,705	0,705	0,749	0,839
porMZ																0,857	0,667	0,185
																0,00178	0,0588	0,619
pgk																	0,5	0,124
																	0,182	0,749
relA																		0,0741
																		0,839

[a] The colour-coding means the following: yellow – 0.5 < r < 0.7, 0.05 > p >0.001, orange: r > 0.7, p<0.001, , abbreviations in Table S1. [b] Metronidazole MIC. [c] Borderline significance

Table S3. Cross-correlation values of gene expressions metronidazole resistance for seven *nim*-negative *B. fragilis* strains[a]

	S3	*acr5*	*acr15*	*crpF*	*frdC*	*feoAB*	*fldA*	*fprA*	*frdA*	*galK*	*gatMZ*	*ldh*	*mdh*	*nanH*	*por*	*pgk*	*relA*	MIC
L20	0,643	-0,786	-0,0357	-0,536	0,821	-0,198	-0,786	0,143	0,679	0,464	-0,018	-0,464	0,643	-0,821	0,0357	0,964	0,536	0,128
	0,0956	0,0251	0,905	0,181	0,0145	0,602	0,0251	0,72	0,0735	0,255	0,905	0,255	0,0956	0,0145	0,905	2E-07	0,181	0,72
S3		-0,107	0,607	-0,25	0,5	-0,577	-0,429	-0,214	0,679	0,857	0,0541	-0,0357	0,25	-0,321	0	0,75	0,5	0,0551
		0,781	0,121	0,545	0,217	0,15	0,297	0,602	0,0735	0,00609	0,843	0,905	0,545	0,438	0,968	0,0384	0,217	0,843
acr5			0,464	0,536	-0,75	-0,018	0,821	-0,179	-0,286	0,0714	-0,234	0,714	-0,679	0,893	0,179	-0,714	-0,214	0,147
			0,255	0,181	0,0384	0,905	0,0145	0,66	0,491	0,843	0,545	0,0545	0,0735	2E-07	0,66	0,0545	0,602	0,72
acr15				0,429	0,143	-0,18	-0,0357	-0,0357	0,5	0,321	0,252	0,571	-0,0357	0,0714	0,321	0,0714	0,464	-0,257
				0,297	0,72	0,66	0,905	0,905	0,217	0,438	0,545	0,15	0,905	0,843	0,438	0,843	0,255	0,545
crpF					-0,143	0,631	0,536	0,0357	0,214	-0,357	0,27	0,857	0	0,464	0,643	-0,607	0,25	0,0551
					0,72	0,0956	0,181	0,905	0,602	0,388	0,491	0,00609	0,968	0,255	0,0956	0,121	0,545	0,843
frdC						0	-0,786	0	0,75	0,143	0,432	-0,321	0,643	-0,857	0,107	0,75	0,5	-0,128
						0,968	0,0251	0,968	0,0384	0,72	0,297	0,438	0,0956	0,00609	0,781	0,0384	0,217	0,72
feoAB							0,252	0,595	0,18	-0,667	-0,0909	0,523	0,324	0,0541	0,775	-0,396	0,36	0,232
							0,545	0,121	0,66	0,0735	0,781	0,181	0,438	0,843	0,0251	0,341	0,388	0,602
fldA								-0,214	-0,393	-0,0714	-0,378	0,607	-0,607	0,964	0,214	-0,821	-0,357	0,496
								0,602	0,341	0,843	0,341	0,121	0,121	2E-07	0,602	0,0145	0,388	0,217
fprA									0,179	-0,393	-0,36	0,25	0,429	-0,286	0,607	0,0714	0,571	-0,0367
									0,66	0,341	0,388	0,545	0,297	0,491	0,121	0,843	0,15	0,905
frdA										0,393	0,0901	0,286	0,607	-0,464	0,607	0,607	0,857	0,239
										0,341	0,781	0,491	0,121	0,255	0,121	0,121	0,00609	0,545
galK											-0,252	-0,0714	0	0,0357	-0,143	0,571	0,214	0,385
											0,545	0,843	0,968	0,905	0,72	0,15	0,602	0,341
gatMZ												-0,144	0,306	-0,306	-0,27	0,0541	-0,018	-0,722[c]
												0,72	0,438	0,438	0,491	0,843	0,905	0,0545
ldh													-0,0714	0,571	0,786	-0,5	0,429	0,257
													0,843	0,15	0,0251	0,217	0,297	0,545
mdh														-0,643	0,357	0,607	0,75	-0,11
														0,0956	0,388	0,121	0,0384	0,781
nanH															0,0714	-0,786	-0,393	0,349
															0,843	0,0251	0,341	0,388
por																-0,107	0,75	0,404
																0,781	0,0384	0,341
pgk																	0,5	-0,0184
																	0,217	0,905
relA																		0,11
																		0,781

[a] The colour-coding means the following: yellow – 0.5 < r < 0.7, 0.05 > p >0.001, orange: r > 0.7, p<0.001, abbreviations in Table S1. [b] Metronidazole MIC.

. [c] Borderline significance

Fig. S1. The tricarboxylic acid pathways of *B. fragilis*

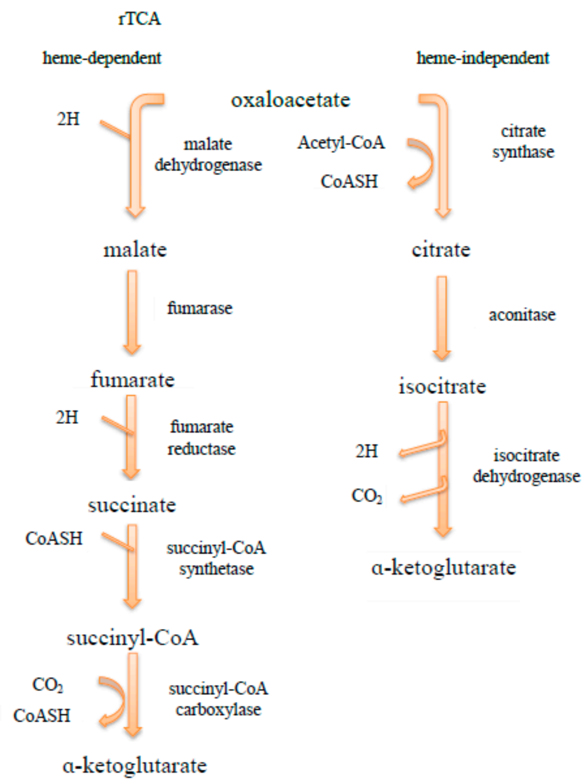

Fig. S2. Examples of the Etest results on modified M9 medium

B. fragilis 638R (A) and *B. fragilis* 638R/*nimA* (B) on glucose-supplemented M9 medium, respectively.

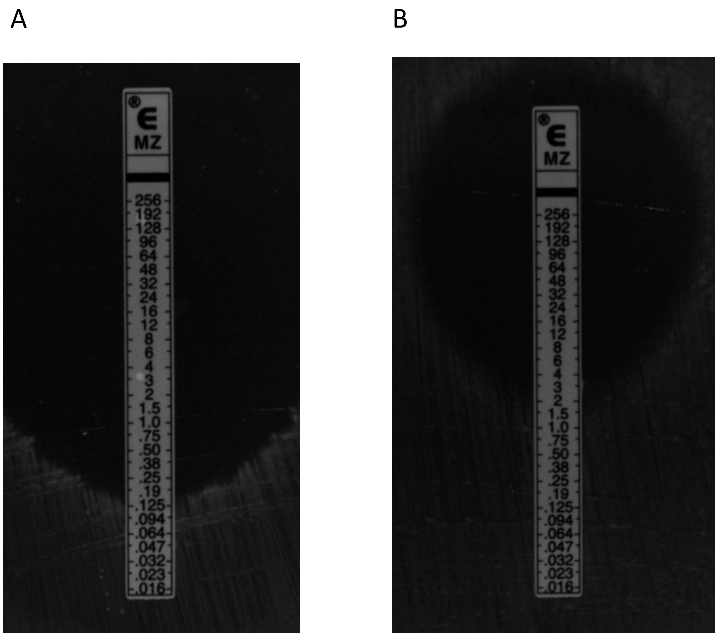

Table S4. Effects of C_4 dicarboxylic acid supplementation on metronidazole MICs of selected *nim*-positive and negative *B. fragilis* strains

Strain	*nim*	Media (MICs in µg/ml)					
		SCA	M9-G[a]	M9-O	M9-M	M9-F	M9-S
638R	-	0.125	0.25	0.064	0.032	0.032	0.032
638R/*nimA*	*nimA*	4	4	4	4	2	4
KSB-R	*nimB*	16	>256[b]	16	256	16	>256
19811	-	32	>256	64	>256	16	>256
18807i2	-	(0.25-)>256[c]	0.5	0.25	0.125	0.125	0.125
Q5	*nimE*	>256	>256	>256	>256	256	>256

[a] M9 media supplemented with glucose (G), oxaloacetic acid (O), malate (M), fumarate (F) and succinate (S).

[b] Significantly increased metronidazole resistance values are highlighted in yellow. [c] Heterogeneous resistance phenotype.
